# Serum- and glucocorticoid-inducible kinase 3 is a potential oncogene in nasopharyngeal carcinoma^☆^

**DOI:** 10.1016/j.bjorl.2018.05.012

**Published:** 2018-07-18

**Authors:** Jing Chen, Hai Liang Li, Bo Bo Li, Wei Li, Dong Ma, Yong He Li, Tao Liu

**Affiliations:** a Zhujiang Hospital, Southern Medical University, Department of Otolaryngology, Guangzhou, Guangdong, China; b Jinan University, Zhuhai People's Hospital, Xiangzhou District, Zhuhai, Guangdong, China; c University of Science and Technology of China, School of Life Sciences and Medical Center, The Chinese Academy of Sciences Key Laboratory of Innate Immunity and Chronic Diseases, Hefei, Anhui, China; d Guangdong Provincial Hospital of Chinese Medicine, Department of Otolaryngology, Guangdong, China; e The 2^nd^ Clinical College of Guangzhou University of Chinese Medicine, Guangzhou, China; f Jinan University, Department of Biomedical Engineering, Key Laboratory of Biomaterials of Guangdong Higher Education Institutes, Guangzhou, Guangdong, China; g Guangdong Academy of Medical Sciences, Guangdong General Hospital, Department of Otolaryngology, Guangzhou, Guangdong, China

**Keywords:** Nasopharyngeal carcinoma, Serum- and glucocorticoid-inducible kinase 3, shRNA, Proliferation, Apoptosis, Migration, Carcinoma nasofaríngeo, Quinase 3 sérica e induzida por glicocorticoide, shRNA, Proliferação, Apoptose, Migração

## Abstract

**Introduction:**

Serum- and glucocorticoid-inducible kinase 3, a serine/threonine kinase that functions downstream of the PI3K signaling pathway, plays a critical role in neoplastic processes. It is expressed by various tumors and contributes to carcinogenesis.

**Objective:**

The objective was to investigate serum- and glucocorticoid-inducible kinase 3 expression in nasopharyngeal carcinoma, to study the anti-tumor effects of serum- and glucocorticoid-inducible kinase 3 shRNA by inhibiting its expression in nasopharyngeal carcinoma cells and to discuss the potential implications of our findings.

**Methods:**

Serum- and glucocorticoid-inducible kinase 3 protein expression in nasopharyngeal carcinoma cell lines (CNE-1, CNE-2, HNE-1, HONE-1, and SUNE-1) and the human immortalized nasopharyngeal epithelium cell line NP69 were assayed by western blotting. Serum- and glucocorticoid-inducible kinase 3 expression in 42 paraffin-embedded nasopharyngeal carcinoma tissues were performed by immunohistochemistry. MTT assay, flow cytometry, and scratch tests were performed after CNE-2 cells were transfected with the best serum- and glucocorticoid-inducible kinase 3 shRNA plasmid selected by western blotting using lipofectamine to study its effect on cell proliferation, apoptosis, and migration.

**Results:**

Serum- and glucocorticoid-inducible kinase 3 was overexpressed in human nasopharyngeal carcinoma tissues and cells. Serum- and glucocorticoid-inducible kinase 3 expression decreased markedly after CNE-2 cells were transfected with the serum- and glucocorticoid-inducible kinase 3 shRNA, leading to strong inhibition of cell proliferation and migration. In addition, the apoptosis rate increased in CNE-2 cells after serum- and glucocorticoid-inducible kinase 3 knockdown.

**Conclusion:**

Serum- and glucocorticoid-inducible kinase 3 expression was more frequently observed as the nasopharyngeal epithelium progresses from normal tissue to carcinoma. This suggests that serum- and glucocorticoid-inducible kinase 3 contributes to the multistep process of NPC carcinogenesis. Serum- and glucocorticoid-inducible kinase 3 represents a target for nasopharyngeal carcinoma therapy, and a basis exists for the further investigation of this adjuvant treatment modality for nasopharyngeal carcinoma.

## Resumo

**Introdução:**

A quinase 3 sérica induzida por glicocorticoide, uma serina/treonina quinase que funciona *downstream* da via de sinalização PI3K, desempenha um papel crítico nos processos neoplásicos. É expressa por vários tumores e contribui para a carcinogênese.

**Objetivo:**

O objetivo foi investigar a expressão de quinase 3 sérica induzida por glicocorticoide no carcinoma nasofaríngeo, estudar os efeitos antitumorais do shRNA da quinase 3 sérica induzida por glicocorticoide, inibindo sua expressão em células de carcinoma nasofaríngeo e discutir as implicações potenciais de nossos achados.

**Método:**

A expressão de proteína quinase 3 sérica induzida por glicocorticoide em linhagens de células de carcinoma nasofaríngeo (CNE-1, CNE-2, HNE-1, HONE-1 e SUNE-1) e a linhagem de células humanas imortalizadas do epitélio nasofaríngeo NP69 foram avaliadas por *Western blot*. A expressão da quinase 3 sérica induzida por glicocorticoide em 42 tecidos de CNF embebidos em parafina foi realizada por imunohistoquímica. Testes com MTT, citometria de fluxo e testes de raspagem foram realizados após as células CNE-2 terem sido transfectadas com o melhor plasmídeo shRNA da quinase 3 sérica induzida por glicocorticoide selecionado por *Western blot*, utilizando lipofectamina para estudar seu efeito na proliferação, apoptose e migração celular.

**Resultados:**

Foi observada uma sobre-expressão da quinase 3 sérica induzida por glicocorticoide em tecidos e células de carcinoma nasofaríngeo humanas. A expressão de quinase 3 sérica induzida por glicocorticoide diminuiu acentuadamente após as células CNE-2 terem sido transfectadas com o shRNA da quinase 3 sérica induzida por glicocorticoide, conduzindo à forte inibição de proliferação e migração celular. Além disso, a taxa de apoptose aumentou nas células CNE-2 após o *knockdown* da quinase 3 sérica induzida por glicocorticoide.

**Conclusão:**

A expressão de quinase 3 sérica induzida por glicocorticoide foi observada com maior frequência à medida que o epitélio nasofaríngeo progride de tecido normal para carcinoma. Isto sugere que a quinase 3 sérica induzida por glicocorticoide contribui para o processo multi-etapas da carcinogênese do carcinoma nasofaríngeo. A quinase 3 sérica induzida por glicocorticoide representa um alvo para a terapia do carcinoma nasofaríngeoe há uma base para a investigação adicional desta modalidade de tratamento adjuvante para o carcinoma nasofaríngeo.

## Introduction

Nasopharyngeal carcinoma (NPC) is one of the most common head and neck tumors and originates from the nasopharyngeal mucosa.^1^ It frequently occurs in southern China and southeast Asia due to its regional distribution and familial aggregation.^2^ NPC is especially common in China, with an incidence of 20/100 000, but extremely rare in western countries, with an incidence of less than 1/100 000.^3^

Most pathological types of NPCs are low-differentiated squamous cell carcinomas, which exhibit a high degree of malignant and cervical lymph node metastasis at an early stage.^4^ There were an estimated 60 600 incident cases of NPCs and 34 100 deaths reported in China in 2015.^5^ Furthermore, the number of new NPC cases is increasing exponentially.

Comprehensive treatment consisting of radiotherapy combined with chemotherapy is currently the main clinical treatment strategy for NPC,^6^ and local recurrence and distant metastasis are the main reasons for the failure of NPC treatment.^7^ The pathogenesis of NPC is not clear, and additional factors may be involved, such as family history, genetic susceptibility, environmental or stochastic factors, and Epstein–Barr virus (EBV) infection.^3^ However, the molecular processes involved in NPC pathogenesis have not yet been well defined. An improved understanding of the molecular mechanisms of NPC progression may contribute to the development of new therapeutic approaches and improve the survival of NPC patients.

Serum- and glucocorticoid-inducible kinases (SGKs) are serine/threonine kinases that function downstream of the phosphoinositide 3 kinase (PI3-K) signaling pathway.^8^ SGKs have three isoforms in mammals (SGK1, SGK2, and SGK3) and belong to the AGC kinase family (protein kinase A, protein kinase G, and protein kinase C), similar to protein kinase B/AKT.^9^ SGKs share great homology in the kinase domain with AKT,^10^ are involved in the PI3-K pathway, and regulate cellular processes similar to AKT. As a member of the SGK family, SGK3 activation and phosphorylation at Thr320 by phosphoinositide-dependent kinase-1 (PDK1) is considered an AKT-independent manner of signaling in cancer.^11^ SGK3 has been suggested to play a pivotal role in the genesis and development of many tumors.^12^, ^13^, ^14^, ^15^, ^16^ However, little attention has been given to its effects in NPC.

In this paper, we explored the expression and potential roles of SGK3 in human NPC. Our studies identify SGK3 as a novel potential promoter of human NPC development and suggest a new therapeutic target for NPC therapy.

## Methods

### Material

The human NPC cell lines (CNE-1, CNE-2, HNE-1, HONE-1 and SUNE-1) and the human immortalized nasopharyngeal epithelium cell line NP69 were preserved by our laboratory. CNE1 is a nasopharyngeal, well-differentiated squamous cell carcinoma cell line; CNE2, HNE1, HONE1 and SUNE1 are nasopharyngeal, poorly differentiated squamous cell carcinoma cell lines; NP69 is an immortalized nasopharyngeal epithelial cell line, showing squamous epithelioid and adherent growth, with normal characteristics of the nasopharyngeal epithelium. The five NPC cell lines were cultured in an RPMI-1640 medium (Gibco, Life Technologies Inc., Carlsbad, CA, USA) containing 10% fetal bovine serum (Tianhang Biotechnology Co., Ltd., Zhejiang, China). NP69 cells were cultured in a keratinocyte/serum-free medium (K-SFM; Gibco) supplemented with bovine pituitary extract (Gibco) and epidermal growth factor (human recombinant; Gibco).

The cells were all cultured at 37 °C with 5% CO_2_. The SGK3 shRNA plasmids were all produced by GeneCopoeia Inc., Rockville, MD, USA, and included sh1025, sh1132, sh1206 and sh1284; the sequences are 5′-GCTAGTGCATTGGGTTACTTA-3′, 5′-GCTTTGTAAAGAAGGAATTGC-3′, 5′-GCCGAGATGTTGCTGAAATGT-3′ and 5′-GGTCCATTCTGGAAGAACTCC-3′, respectively. The negative control (NC) plasmid, with a sequence 5′-GCTTCGCGCCGTAGTCTTA-3′, was also purchased from GeneCopoeia Inc. To obtain the best sequence of the plasmid on gene silencing, the cells were seeded before they were transfected and allowed to grow to a density of 60% on the second day. The cationic polymer PEI (obtained from Aladdin Inc.) was used to transfect the plasmids with different sequences of ShRNA (silent different locus of SGK3) into cells. After 72 h, cell proteins were extracted, and western blotting was used to observe the silencing effects of ShRNA on SGK3, and select the plasmid with the best sequence on silencing. The best sequence is 5′-GCTAGTGCATTGGGTTACTTA-3′.

### Main equipment

A carbon dioxide incubator was produced by Thermo Fisher Scientific Inc., USA. An HM340E-rotary slicer and a hematoxylin–eosin (HE) staining fully automatic dyeing machine were produced by MICROM Inc., Germany. A vertical electrophoresis apparatus and transmembrane system were produced by Bio-Rad Inc., Hercules, California, USA. The ChemiDoc™ XRS + chemiluminescence imaging system was also produced by Bio-Rad Inc. The BD FACSCalibur™ flow cytometer was produced by BD biosciences Inc., California, USA. An inverted fluorescence microscope was produced by Carl Zeiss Co. Ltd., Germany.

### Immunohistochemistry

Forty-two NPC paraffin-embedded tissue samples and nine chronic nasopharyngitis tissue samples were obtained from the Department of Otolaryngology of Zhujiang Hospital. The clinicopathological data is presented in Table 1, Table 2. The SGK3 expression in sections was assayed via immunohistochemistry with the Elivision two-step detection kit (Kit-0015; Fuzhou Maixin Biotech. Co., Ltd., China). These sections were deparaffnized and pretreated by boiling the slides in citrate buffer (pH 6) for 10 min. The sections were then immersed in 3% hydrogen peroxide for 10 min at room temperature to block endogenous tissue peroxidase activity. Following washing with phosphate-buffered saline (PBS), sections were incubated with Rabbit SGK3 polyclonal antibody (catalog no. ab153981; 1:200; Abcam Company, USA) at 4 °C overnight, and then sections were incubated with 50 μL amplifer polymer (reagent A), and 50 μL horse radish peroxidase-conjugated anti-mouse/rabbit IgG (reagent B) for 30 min at room temperature. Following washing with PBS, the sections were stained with diaminobenzidine chromogenic solution (DAB) and counterstained with hematoxylin, differentiated using hydrochloric acid in ethanol, blued by washing with water and sealed with conventional resin.^17^ The results were evaluated by the HSCORE method which included both the intensity and the distribution score of the specific staining, evaluated by two pathologists independently.^18^ The distribution scores of SGK3 expression were recorded as percentages of positively stained cells in each of the four intensity categories, which were scored as 0 (no staining), 1 (weak staining), 2 (distinct staining) and 3 (strong staining). The HSCORE of each tissue was derived by summing the four distribution scores (*D*) of each category multiplied by the intensity scores (i) as follows: HSCORE = Σ*D*(*i*), where *D* varied from 0 to 100% and *i* = 0, 1, 2 or 3. The maximum of HSCORE was 300, and the minimum was 0. An HSCORE = 75 was used as the cut-off point to distinguish positive and negative expression.

**Table 1:** Clinicopathological characteristics of NPC patients and tissues.

		NPC cases (positive rate, %)
Gender	Male	31 (73.8%)
	Female	11 (26.2%)
Age	<55	30 (71.4%)
	≥55	12 (28.6%)
T stage	T1	8 (19.0%)
	T2	10 (23.8%)
	T3	13 (31.0%)
	T4	11 (26.2%)
N stage	N0	6 (14.3%)
	N1	13 (31.0%)
	N2	15 (35.7%)
	N3	8 (19.0%)
M stage	MO	38 (90.5%)
	M1	4 (9.5%)
Clinical stage	Stage I	6 (14.3%)
	Stage II	9 (21.4%)
	Stage III	15 (35.7%)
	Stage IV	12 (28.6%)

**Table 2:** Comparison of gender and age between NPC and chronic nasopharyngitis patients.

Group	*n*	Gender		*χ*^2^	*P*	Age	*t*	*P*
		Male	Female					
NPC tissues	42	31	11			50.0±11.3		
				2.968	0.085		1.043	0.312
Chronic nasopharyngitis tissues	9	4	5			39.4 ± 13.4		

This study was approved by the Ethics Committee (no. 2016-EBYHK-002).

### Western blot analysis

Total proteins were extracted from cells using RIPA lysis buffer (Kaiji Biotechnology Co., Ltd., Jiangsu, China). Protein concentrations were determined using protein assays (Beyotime Biotechnology Co., Ltd., Shanghai, China). Purified proteins (50 μL) were separated by sodium dodecyl sulfate–polyacrylamide gel electrophoresis (SDS–PAGE) and transferred to polyvinylidene difluoride membranes (Millipore, Bedford, MA, USA). The membranes were blocked for 2 h at room temperature in 5% non-fat milk and incubated overnight at 4 °C with an anti-SGK3 antibody (Abcam Company, 1:1000). Following incubation with a horseradish peroxidase (HRP), linked secondary antibody (Biosharp Biotechnology Co., Ltd., Hefei, China) (1:5000), the membranes were washed, and proteins were visualized using an enhanced chemiluminescence kit (PerkinElmer Inc., Waltham, MA, USA). Western blotting results were semi-quantitatively analyzed by Image-Pro Plus software.^19^

### Plasmid transfection and screening

Logarithmic phase CNE-2 cells were seeded in 6-well plates for 24 h before transfection to achieve 80–90% confluence. Cells in each well were transfected with 4 μg of SGK3 shRNA or NC plasmid and 10 μL of Lipofectamine 2000 (Invitrogen, Life Technologies Inc., Carlsbad, California, USA). Non-treated cells were used as the control. All cells were cultured at 37 °C for 24 h. After determining the success of transfection according to the degree of green fluorescent protein (GFP) by fluorescence microscopy, the cells were cultured for another 48 h and harvested for western blot analysis to identify the plasmid with the greatest effect on SGK3 knockdown.^20^ The treated cells were harvested for subsequent experiments. Cells successfully transfected with the SGK3 shRNA or NC plasmid were referred to as the shSGK3 or NC group, respectively. CNE-2 cells served as a blank control (control group). All in vitro experiments were performed for each group.

### Growth rate assay

CNE-2 cells in the three groups were cultured in 96-well plates (5000 cells/well in five wells). Cell growth was examined using a 3-(4,5)-dimethylthiahiazo(-z-y1)-3,5-di-phenytetrazoliumromide (MTT) assay with one plate conducted at the indicated time-points for three consecutive days. Each well contained 20 μL of MTT reagent and was then cultured at 37 °C for 4 h. The medium was discarded, and crystals that had formed were dissolved in 150 μL of dimethyl sulfoxide (DMSO). The viable cells in each well were determined each day by measurement at an absorbance wavelength of 490 nm. Each experiment was repeated independently three times. Cell growth curves were subsequently generated.^20^

### Cell-cycle analysis

CNE-2 cells in the three groups were seeded in 6-well plates (50 000 cells/well) and cultured for 48 h. At the end of incubation, all cells were trypsinized, washed twice with pre-cooled PBS (HyClone, Logan, UT, USA), collected and resuspended in 100 μL of binding buffer. Then, 5 μL of annexin V-APC and 5 μL of PI were added, and the plates were incubated for 30 min in the dark at room temperature. Cells were immediately examined by the flow cytometer. Data were analyzed by ModFit LT 2.0 software.^21^

### Scratch tests

CNE-2 cells were added to 6-well plates (50 000 cells/well). Once the cells had grown to approximately 100% confluence, scratch tests were performed using a 200 μL sterile pipette tip to scratch the confluent monolayer. The scratched area was washed with PBS until the detached cells were removed. Serum-free medium was added, and the cells were cultured at 37 °C. The distance between the cells in the scratched area at 0, 24 and 48 h was measured by microscopy.^22^ Horizontal cell migration was analyzed by Image J software and calculated using the following formula: the relative mobility = 1 (immediate scratch width/original scratch width).

### Statistical analysis

Statistical evaluation was performed using SPSS 22.0 or Graph Pad Prism 6 software. All data were acquired from at least three independent experiments, and the results are presented as the mean ± standard deviation. One-way analysis of variance and Student's *t*-tests were used to compare the differences in means among groups.

The association of SGK3 expression with the clinical features in NPC was assayed by logistic regression analysis. A value of *p* < 0.05 indicated a significant difference.

## Results

### SGK3 expression in NPC tissues

**Figure 1:**
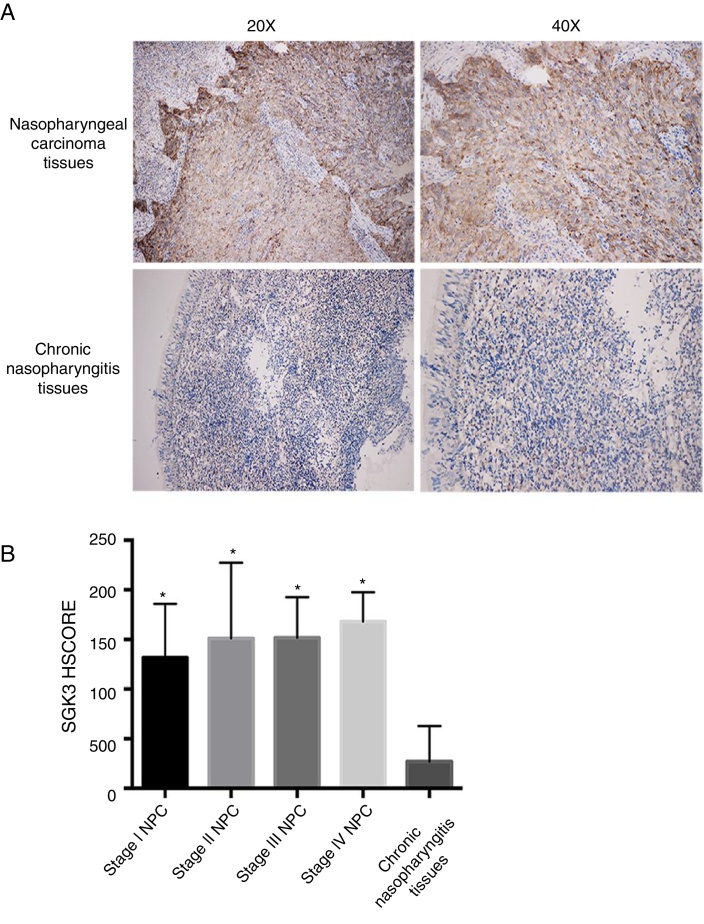
Representative immunohistochemical staining of SGK3 in NPC tissues. (A) Immunohistochemical results showed that SGK3 protein expression was mainly located in the cytoplasm, with strongly positive SGK3 expression in NPC cancer nests and no expression or weak expression in chronic nasopharyngitis tissues. (B) SGK3 expression rate in different stages of NPC and chronic nasopharyngitis tissues according to the HSCORE. The SGK3 expression in stage I, II, III and IV NPC tissues was significantly higher than that in chronic nasopharyngitis tissues (*p* < 0.01). However, no significant difference was observed among the different NPC stages (*p* > 0.05).

Fig. 1A shows SGK3 protein expression in tissues. The positive expression rates of SGK3 in NPC tissues and chronic nasopharyngitis tissues were 90.50% (38/42) and 11.10% (1/9), respectively. The SGK3 expression rate at different NPC stages and in chronic nasopharyngitis tissues was assessed according to the HSCORE.

The SGK3 expression level was significantly higher in NPC than in chronic nasopharyngitis tissue (*p* < 0.01) (Fig. 1B), indicating that SGK3 expression is related to NPC carcinogenesis. However, SGK3 expression was not correlated with gender, age, TNM stage or clinical stage (*p* > 0.05) (Table 3).

**Table 3:** Correlation of SGK3 expression in NPC tissues (*n* = 42).

	*n*	SGK3		*P*
		Positive expression (Positive rate, %)	Negative expression	
*Gender*				
Male	31	29 (93.55%)	2	0.242
Female	11	9 (81.82%)	2	
*Age*				
<55	30	27 (90.0%)	3	0.633
≥55	12	11 (91.67%)	1	
*T stage*				
T1	12	9 (75.0%)	3	0.478
T2	3	3 (100.0%)	0	
T3	16	15 (93.75%)	1	
T4	11	11 (100.0%)	0	
*N stage*				
N0	6	5 (83.33%)	1	0.765
N1–3	36	33 (91.67%)	3	
M Stage				
M0	39	35 (89.74%)	4	0.500
M1	3	3 (100.0%)	0	
*Clinical stage*				
Stage I	6	5 (83.33%)	1	0.536
Stage II	9	7 (77.78%)	2	
Stage III	15	14 (93.33%)	1	
Stage IV	12	12 (100.0%)	0	

### SGK3 expression in NPC cells

**Figure 2:**
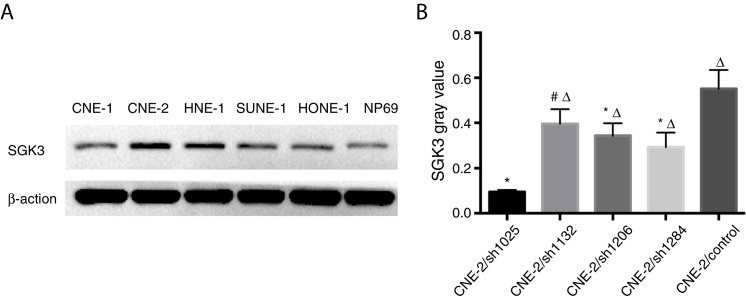
Detection of SGK3 protein expression in NPC cell lines. (A) Representative SGK3 protein expression in different NPC cell lines and NP69 cells detected by western blot analysis. (B) Analysis of the gray value of SGK3 protein expression as the ratio of SGK3 to β-actin in the western blot results; SGK3 was more highly expressed in most NPC cell lines (CNE-2, HNE-1, SUNE-1) than in NP69 cells (*p* < 0.01).

Expression of the SGK3 protein in different NPC cell lines and NP69 cells was as shown in Fig. 2. SGK3 was more highly expressed in most NPC cells (CNE-2, HNE-1, SUNE-1) than in NP69 cells (*p* < 0.01). Moreover, CNE-2 and HNE-1 cells exhibited higher levels of SGK3 expression than other NPC cell lines (*p* < 0.01). The results of the western blot analysis were consistent with those of the immunohistochemistry analysis. These results also indicated that SGK3 might be closely related to NPC development.

### In vitro transfection and plasmid screening

**Figure 3:**
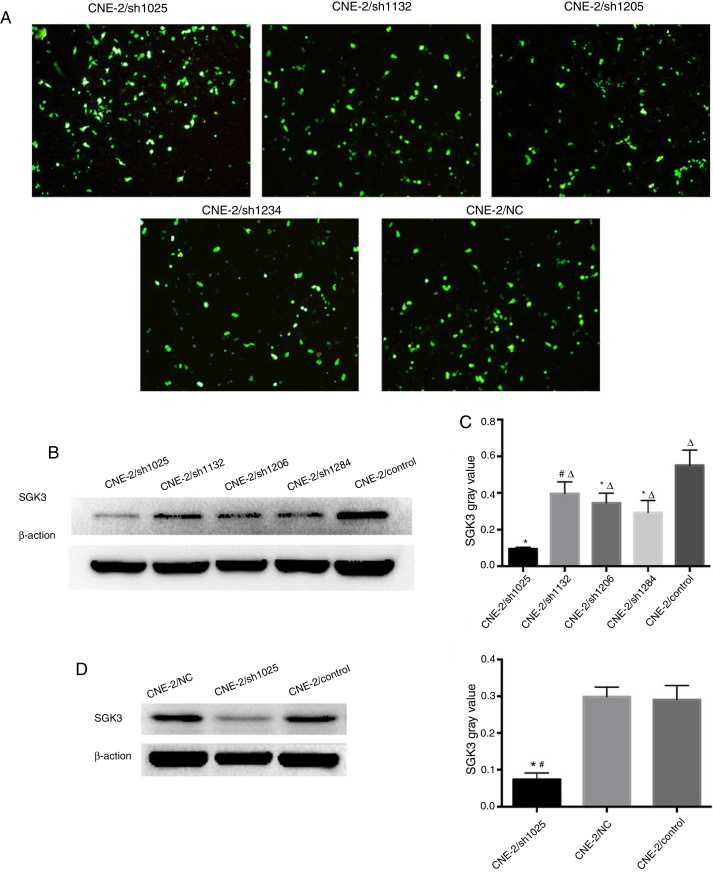
The best SGK3 shRNA screening and efficiency of SGK3 silencing by the specific shRNA in CNE-2 cells. (A) GFP expression in CNE-2 cells observed by fluorescence microscopy after transfection with the SGK3 shRNA or NC plasmid. (B) Representative SGK3 protein expression in CNE-2 cells treated with different SGK3 shRNA plasmids determined by western blot analysis. (C) Analysis of the gray value of SGK3 protein expression as the ratio of SGK3 to β-actin in western blot results. The sh1025 plasmid had the most significant effect on SGK3 knockdown (*p* < 0.01). (D) Representative SGK3 protein expression in shSGK3, NC and control groups determined by western blot analysis. (E) Analysis of the gray value of SGK3 protein expression as the ratio of SGK3 to β-actin in western blot results; expression of SGK3 in the shSGK3 group was obviously reduced compared with that in the NC and control groups (*#*p* < 0.01).

SGK3 shRNA and NC plasmids were successfully transfected into CNE-2 cells (Fig. 3A). The screening results of the most effective shRNA plasmid targeting SGK3 (shSGK3) are presented in Fig. 3B, which shows that the SGK3 protein was notably reduced in the sh1025 group compared with the other plasmid and control groups (*p* < 0.01) (Fig. 3B and C). The sh1025 plasmid had the most significant effect on SGK3 knockdown; therefore, sh1025 group cells were referred to as the hSGK3 group and used in subsequent experiments. The western blot results showed that the SGK3 protein level was markedly reduced in the shSGK3 group compared with that in the NC group and control group (*p* < 0.01) (Fig. 3D and E), and no significant difference in SGK3 expression was observed between the NC group and the control group.

### MTT assay

**Figure 4:**
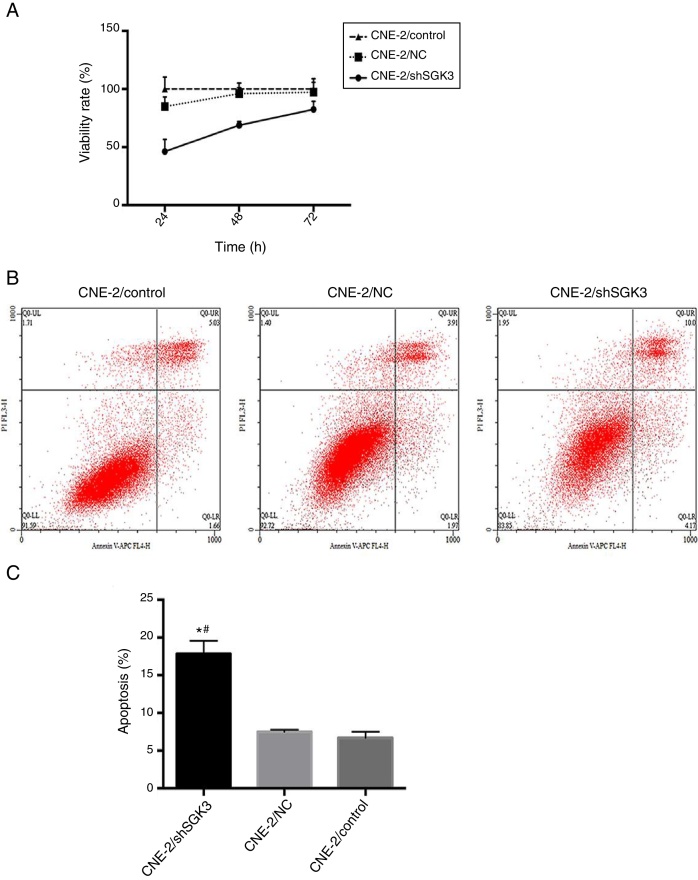
The effect of SGK3 downregulation on the proliferative ability and apoptosis of CNE-2 cells. MTT assay (A) and apoptosis analysis (B, C) by flow cytometry in the different groups. The cell viability rate in the shSGK3 group was obviously reduced compared with that in the NC and control groups, and the cell apoptosis rate in the shSGK3 group was obviously increased compared with that in the NC and control groups (*#*p* < 0.01).

To confirm the cell inhibition effect of SGK3 shRNA, we evaluated its cytotoxic effects. Fig. 4A shows the lack of toxicity in the NC group and obvious cytotoxicity in the shSGK3 group at 24, 48 and 72 h (*p* < 0.01).

### Apoptosis assay

To determine whether SGK3 shRNA could effectively induce cell apoptosis, we determined the percentage of cell apoptosis after the CNE-2 cells were transfected with SGK3 shRNA. As shown in Fig. 4B, CNE-2 cells displayed limited apoptosis in the NC group compared with that in the control group, which was consistent with the results of the cytotoxicity analysis. The percentages of cell apoptosis (including early and late apoptosis) in the shSGK3 group, the NC group and the control group after 48 h were 17.85, 7.49 and 6.70%, respectively. The cells treated with SGK3 shRNA exhibited a more obvious increase in apoptosis (*p* < 0.01) (Fig. 4C).

### Cell scratch test

**Figure 5:**
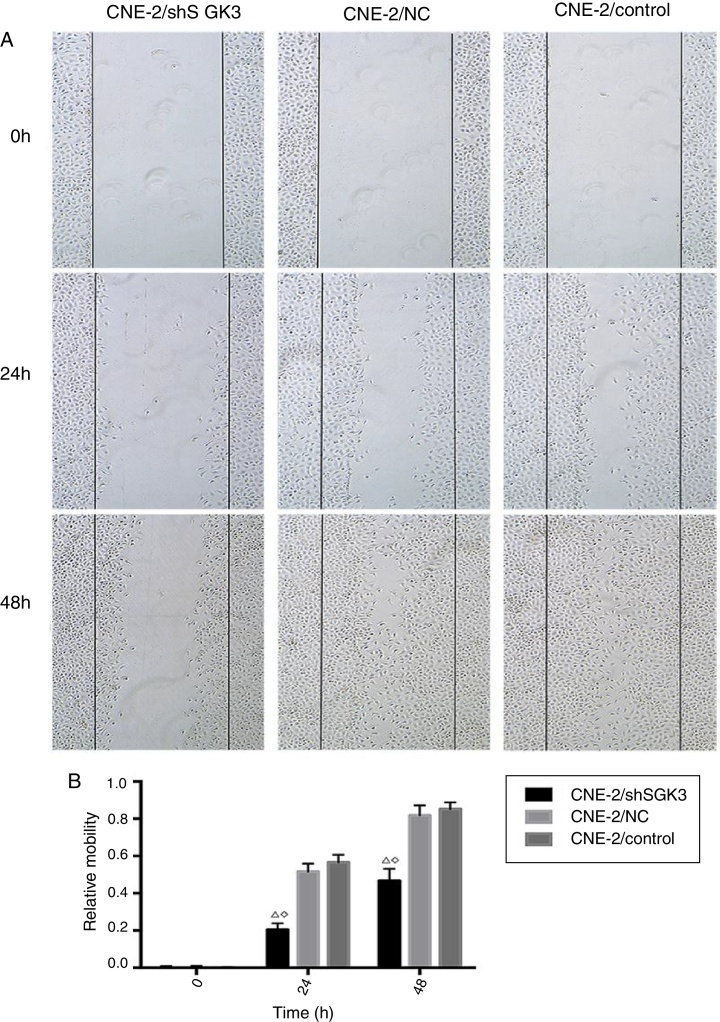
The effect of SGK3 downregulation on the motility of CNE-2 cells. (A) Distance at different times after the scratch test (original magnification: ×5). Scratch distances in the shSGK3 group did not obviously change after 24 h, whereas cells in the NC and control groups exhibited higher migration rates (*p* < 0.01). After 48 h, cells in the shSGK3 group had not yet recovered from the damage (*p* < 0.01), but cell scratches in the NC and control groups were hardly visible. (B) Comparison of the cell migration rates at different times by the scratch test; the cell migration rates in the shSGK3 group were higher than those the NC and control groups after 24 and 48 h (△◊ *p* < 0.01).

To detect the invasion and metastasis ability of tumor cells, the cell scratch test was conducted in the control group, NC group and shSGK3 group. As shown in Fig. 5A, the horizontal cell migration rate in the shSGK3 group after 24 h was 20.50%, whereas these rates were 51.60 and 56.60% in the NC group and control group, respectively. After 48 h, cells in the shSGK3 group had not yet recovered from the damage. The horizontal cell migration rate was 46.70%, but the scratches in the NC group and control group were hardly visible. These results suggested that the migratory ability of the shSGK3-treated cells was obviously decreased compared with that in the NC group and the control group (*p* < 0.01) (Fig. 5B).

## Discussion

The development and progression of tumors are complex biological processes involving a number of genes and molecular pathways. Abnormal activation of the PI3K/AKT pathway and its downstream targets have been observed in a wide range of human malignancies, including NPC.^23^, ^24^, ^25^ SGK3 is a serine/threonine kinase that functions downstream of PI3K, shares both sequence and functional similarity with the AKT family, and plays a critical role in AKT-independent oncogenic signaling.^26^ Although many studies show that SGK3 plays an important role in the regulation of cell proliferation and migration,^27^, ^28^, ^29^, ^30^, ^31^ the functions of SGK3 in human NPC remained unknown.

Liu found that SGK3 overexpression was significantly associated with a poor prognosis and more common than AKT overexpression in hepatocellular carcinoma.^32^ Moreover, Xu studied SGK3 expression in 1340 human breast tumors and found that SGK3 plays an important role in AKT-independent oncogenic signaling.^33^ Another study showed only 36% SGK3 activation was detected in a panel of ovarian tumor samples presenting with low levels of phosphorylated AKT but with high levels of PIK3CA, an encoding gene of P110 alpha subunit in class I PI3-K, and no correlation was found between SGK3 phosphorylation and phosphorylated PIK3CA overexpression or AKT activation.^34^

In the study, we also found higher expression of SGK3 in human NPC tissues and cells and SGK3 expression was not correlated with gender, age, TNM stage or clinical stage. SGK3 expression had no significant correlation with NPC prognosis, suggesting that SGK3 is likely not implicated in aberrant PI3-K oncogenic signaling. However, the number of samples in our study was not sufficient; larger patient cohorts in future studies are required to clarify the role of SGK3 in aberrant PI3-K oncogenic signaling.

As described above, SGK3 plays an important role in the regulation of cancer cell proliferation and migration. Research has shown that dihydrotestosterone (DHT) can upregulate SGK3 expression via androgen receptor (AR) and promote the proliferation of prostate cancer cells.^35^ Sun found that SGK3 expression could promote the proliferation, survival, invasion and migration of breast cancer cells.^36^ A functional in vitro experiment showed that overexpression of SGK3 could promote cell growth, clonogenicity and anchorage-independent growth in hepatocellular carcinoma.^32^ In contrast, knockdown of SGK3 could significantly inhibit these cell processes. Moreover, the study also found that overexpression of SGK3 in hepatocellular carcinoma cells significantly increased tumor formation and progression in nude mice compared to the use of empty vector control cells.

Due to the high expression levels of SGK3 in NPC tissues and cells, and the functions of SGK3 in the cell process, we further explored the effects of SGK3 in NPC cells. We successfully silenced SGK3 expression in NPC cells. The transfection results were verified by western blot analysis of different groups of cells. Growth rate assays revealed that cells in the shSGK3 group had weaker proliferation rates in the same logarithmic phase than those in the NC and control groups. In addition, SGK3-silenced cells presented higher rates of apoptosis than the NC and control group cells. Moreover, scratch tests verified that NPC cells in the shSGK3 group were less resistant to external damage, and their migratory ability was obviously decreased. These results indicated that expression of exogenous SGK3 might alter the behavior of NPC cells and significantly improve NPC cell multiplication and invasion.

## Conclusion

Our study demonstrates that SGK3 is overexpressed in human NPC tissues and cells and that SGK3 silencing could suppress proliferation, survival and migration of NPC cells. It indicated that SGK3 might contribute to NPC pathogenesis. SGK3 may be a potential marker and an efficacious therapeutic target in human NPC.

## Funding

This work was financially supported by the National Natural Science Foundation of China (no. 81573000, 81260406 and 51573071).

## Conflicts of interest

The authors declare no conflicts of interest.
